# Molecular chaperones and selection against mutations

**DOI:** 10.1186/1745-6150-3-5

**Published:** 2008-02-26

**Authors:** Katarzyna Tomala, Ryszard Korona

**Affiliations:** 1 Institute of Environmental Sciences, Jagiellonian University, Gronostajowa 7, 30-387 Krakow, Poland

## Abstract

**Background:**

Molecular chaperones help to restore the native states of proteins after their destabilization by external stress. It has been proposed that another function of chaperones is to maintain the activity of proteins destabilized by mutation, weakening the selection against suboptimal protein variants. This would allow for the accumulation of genetic variation which could then be exposed during environmental perturbation and facilitate rapid adaptation.

**Results:**

We focus on studies describing interactions of chaperones with mutated polypeptides. There are some examples that chaperones can alleviate the deleterious effects of mutations through increased assistance of destabilized proteins. These experiments are restricted to bacteria and typically involve overexpression of chaperones. In eukaryotes, it was found that the malfunctioning of chaperones aggravated phenotypic aberrations associated with mutations. This effect could not be linked to chaperone-mediated stabilization of mutated proteins. More likely, the insufficient activity of chaperones inflicted a deregulation of multiple cellular systems, including those responsible for signaling and therefore important in development. As to why the assistance of mutated proteins by chaperones seems difficult to demonstrate, we note that chaperone-assisted folding can often co-exist with chaperone-assisted degradation. There is growing evidence that some chaperones, including those dependent on Hsp90, can detect potentially functional but excessively unstable proteins and direct them towards degradation instead of folding. This implies that at least some mutations are exposed rather than masked by the activity of molecular chaperones.

**Conclusion:**

It is at present impossible to determine whether molecular chaperones are mostly helpers or examiners of mutated proteins because experiments showing either of these roles are very few. Depending on whether assistance or disposal prevails, molecular chaperones could speed up or slow down evolution of protein sequences. Similar uncertainties arise when the concept of chaperones (mostly Hsp90) as general regulators of evolvability is considered. If the two roles of chaperones are antagonistic, then any (even small) modification of the chaperone activities to save mutated polypeptides could lead to increased misfolding and aggregation of other proteins. This would be a permanent burden, different from the stochastic cost arising from indiscriminate buffering of random mutations of which many are maladaptive.

**Reviewers:**

This article was reviewed by A. S. Kondrashov, J. Höhfeld (nominated by A. Eyre-Walker) and D. A. Drummond (nominated by C. Adami). For the full reviews, please go to the Reviewers' comments section.

## Background

The phenotype often remains stable in spite of extensive variation in the genetic material [1,2]. This has been demonstrated in experiments in which many genes were deleted [3-6] or mutated [7,8]. It has been proposed that such genetic robustness may be an adaptation because it reduces phenotypic penetration of deleterious mutations [9,10]. This concept is still under debate and its full comprehension will require detailed study of the underlying mechanisms [11]. It has been suggested that molecular chaperoning may be one of these mechamisms [12]. Chaperoning is carried out by molecules originally denoted as heat shock proteins (Hsps). They are especially abundant in cells subject to environmental stress in which they help to reactivate destabilized and/or aggregated proteins [13-16]. Most chaperones are also present in normally growing cells [17,18]. Under these conditions, random misfolding is especially likely to affect proteins that mutated in such a way as to reduce their structural stability. Regardless of whether the source of damage is environmental or mutational, its effect on a protein is similar. Hydrophobic regions of a polypeptide, normally buried inside the protein, become exposed on its surface. This is a signal of protein damage, recognized universally by a variety of molecular chaperones [19,20]. Through binding and release of the destabilized proteins, the chaperones perform a dual action: they ameliorate the danger that proteins will aggregate via the hydrophobic patches and promote the refolding of proteins to their native structures [21-24]. It seems straightforward to assume that because the protein signal and the chaperone reaction are universal, the overall effect of chaperoning is also universal. That is, since the chaperones are known to maintain activity of proteins affected by stress, they should also support functioning of proteins destabilized by mutation. The notion that molecular chaperones are potent and general agents of genetic robustness has gained wide recognition [11]. Its experimental corroboration has been sought in studies involving both bacteria [25-27] and eukaryotes [12,28]. In general, these experiments show that abnormal phenotypes become more evident when cellular levels of chaperones decline. However, such results only hint at the possibility that the observed phenotypic changes result from the poor performance of mutated proteins when the required (re)folding activity of chaperones is insufficient [12,25-28]. An alternative explanation is that malfunctioning of the molecular chaperones leads to malfunctioning of many non-mutated proteins [29]. As it is explained later in more detail, the inflicted epistatic effects can match or even exceed those associated with the mutations destabilizing proteins. Therefore, it is necessary to discriminate carefully between these two different aspects of chaperone activity.

A reevaluation of the role of chaperones in evolution appears unavoidable in light of key developments in research on molecular and cellular functions of these proteins. Over the two last decades, they were found to participate in a wide spectrum of cellular processes. Molecular chaperones are known to assist folding of both nascent and nearly mature proteins, as well as protein translocation, remodeling, secretion, and degradation [30]. Although chaperones engaged in these processes are diverse and often non-homologous, they all recognize their protein substrates in a generally similar way, i.e. by direct binding to hydrophobic surfaces. However, under normal cellular metabolism the binding of a destabilized protein by a chaperone is often only the first step in its further processing. The polypeptide can then be released and allowed to continue its folding. However, the chaperones may also stop or revert folding of a protein to enable its translocation or modification [30]. A particularly intriguing possibility is that some of the most abundant chaperones recognize excessively unstable proteins and direct them towards degradation [31,32]. Originally discovered as agents devised specifically to rescue misfolded proteins, chaperones were then found to participate actively in quality control and disposal of troubled chains in order to prevent toxic interactions among themselves and with other cellular elements. In this perspective, the chaperones are helpers at the time of stress, but can become examiners at the time of normal metabolism, While this is not new for molecular biologists (recently reviewed in [33-36]), it has not been considered in relation to phenotypic masking and genetic robustness [12,28,29,37].

Central to this article will be the question of how the activity of chaperones affects the strength of purifying selection. We accept the opinion that molecular chaperones have a generally stabilizing effect on genetic networks and that mutations are more likely buffered when the networks are robust. Our focus will be on direct interactions between the chaperones and mutated proteins. In particular, we ask when and how the action of molecular chaperones can be diverted from rescue to disposal of destabilized proteins and whether this may influence the rate and mode of protein evolution.

## Molecular chaperones and phenotypic buffering in bacteria

**Figure 1:**
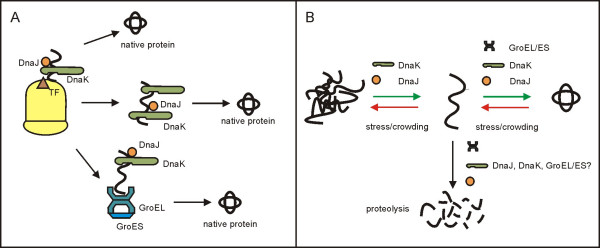
**(A) Chaperone assisted folding of nascent and newly synthesized proteins in bacteria [34].** An elongating polypeptide is initially shielded by TF (trigger factor), a chaperone attached near the exit of the ribosomal tunnel. DnaJ (bacterial Hsp40) initiates binding of the polypeptide by DnaK (Hsp70) during translation. The initial, co-translational assistance of these chaperones is sufficient for the folding of many proteins (upper path). Other proteins are chaperoned by DnaK also after they detach from the ribosome (middle path). There are also proteins whose post-translational folding is assisted by DnaK and then by the GroEL/ES system. **(B) **Chaperone assisted reactivation of secondarily misfolded bacterial proteins. An unfolded protein can be rescued or directed to proteolysis. Participation of the molecular chaperones in recruiting single chains from aggregation and then in their correct folding is well documented. Possible cooperation of the chaperones with proteases has also been reported (see the text for more details). In bacteria, folding of both the primarily and secondarily unfolded proteins is assisted by the same chaperones.

Thermally induced instability is only one possible cause of protein misfolding. Others include overcrowding, transcriptional and translational errors, absence of necessary post-translational partners for proteins, and different chemical and physical stresses. Overcrowding seems to be especially important and universal because the density of macromolecules (proteins and RNAs) within cells is very high, typically about 300 g/l [38]. These conditions necessarily promote frequent misfolding and aggregation of polypeptides [39], especially in regions of intense translation because elongating chains remain largely unfolded until synthesis of a whole protein domain is finished [40]. Translational errors represent another important factor promoting misfolding because they are inherent to biosynthesis and occur at a considerably high rate, between 1/1,000 and 1/10,000 per codon, which may lead to improper amino acids sequences in up to 20% of polypeptides [41]. Bacteria have evolved a chaperone system that assists folding of nascent proteins. Its three major constituents iclude TF, DnaK, and GroEL (Fig. 1A). TF is bound to the ribosome and provides initial shielding to an elongating polypeptide [42]. DnaK is a typical Hsp70 chaperone that recognizes short stretches of hydrophobic amino acids which are expected to occur at multiple positions of most proteins [19]. Individually acting molecules of DnaK bind and release polypeptides in a cycle governed by its two cofactors, DnaJ and GrpE [43]. This helps to unwind and separate intertangled molecules and in this way facilitates proper folding. The Hsp60 type chaperone GroEL works in a different way. It oligomerizes into heptamers forming ring-shaped cages with hydrophobic apical regions. These recognize the hydrophobic parts of substrates which are then, with the help of a smaller chaperone partner GroES, translocated to the interior where their folding can be completed without danger of interference by other proteins [20]. DnaK and GroEL bind not only the nascent proteins but also those secondarily misfolded and aggregated, both under normal metabolism and stress [44-46], (Fig. 1B). They are always abundant and taken together form the core of the bacterial chaperone machinery [24]. The bacterial cell also hosts other chaperones but these two are most likely to be involved in phenotypic buffering of genetic variation.

Elevated levels of the GroEL type chaperone are found in the endosymbiotic bacteria *Buchnera*. It has been suggested that this is an adaptation to a high incidence of mutational destabilization of proteins in these bacteria. They live inside cells in small populations, making natural selection inefficient in removing slightly deleterious mutations from protein coding genes [25]. To test this hypothesis, laboratory populations of *E. coli *with experimentally accumulated multiple deleterious mutations were used. It was found that the mutated clones grow better under induced overexpression of the chaperones GroEL or DnaK [26]. In another experiment involving *E. coli*, mutations were gradually accumulated. Clones that survived had enhanced levels of DnaK and GroEL [27]. The latter finding is especially interesting, because it suggests that an increase in mutational load selects for a concurrent increase in the cellular level of chaperones. However, among the tens or even hundreds of mutations involved in this and the two former studies, there must be some that reduced the level of expression and not the structural stability of proteins. Lowered levels of these proteins could create rate limiting steps in cellular metabolism. Assuming that some of these proteins require chaperones for maturation or remodeling [47,48], increased levels of the chaperones would probably be advantageous. Hypothetically, this, and not the need to support structurally destabilized proteins, could be the reason why natural selection favors high levels of chaperones. Another interesting result was obtained in a study in which an induced overexpression of GroEL resulted in suppression of single heat-sensitive mutations, but not cold-sensitive or temperature insensitive ones [49]. Because heat sensitivity typically accompanies protein instability, this result is close to demonstrating that chaperones can restore the functionality of mutationally destabilized proteins and thus provide means for phenotypic masking in its clearest form. Still better evidence for this comes from experiments with temperature sensitive mutants of the coat protein of the phage P22. Mutated protein is unstable and tends to misfold and aggregate. It was explicitly shown that GroEL/S interacted in vivo with the mutated proteins significantly more intensely than with wild-type protein. Normal levels of the chaperone were sufficient to save most of the mutated protein from aggregation at low temperatures. At standard and higher temperatures, aggregation was curbed by artificial overexpression of the chaperone [50]. This example shows that there are proteins which become dependent on molecular chaperones after mutation and suggests that bacterial cells may increase their buffering capabilities by increasing the cellular level of molecular chaperones. At this point, it is appropriate to ask how the upregulation of chaperones may affect the functioning of other, non-mutated elements of cellular metabolism.

Molecular chaperones are often viewed as competitors to proteases as both of these classes of enzymes recognize destabilized proteins. Normally more abundant, the chaperones are likely to bind transiently most structurally defective proteins and promote their folding, thereby preventing degradation [51]. Interactions with the chaperones would be important for the mutated proteins, especially if they extend over multiple rounds of binding and release, because this could salvage them from proteases. However, even if activity of bacterial chaperones strictly opposes that of proteases, it is still possible that under normal growth the quality control system is balanced at a level of sensitivity appropriate for folding of wild-type but not mutated proteins. Indeed, at least some mutationally destabilized proteins are rapidly degraded under normal cellular concentration of DnaK [52]; recall that stabilization of mutated proteins was observed when chaperones were overexpressed [49]. Moreover, there is evidence that a bacterial chaperone (DnaJ) can aid bacterial proteases (Lon and ClpAP) in the degradation of a mutationally destabilized protein and that this support is not restricted to maintaining the protein in a soluble state [53] but is likely to take more active forms [54]. Similarly, there is indication that if GroEL fails to catalyze the proper folding of a protein, it can facilitate its rapid degradation [55]. Overexpression of molecular chaperones is likely to interfere with protein degradation. Moreover, overexpressed chaperones change the range of proteins with which they interact [56]. Thus, it is likely that permanent change in the cellular level of chaperones may result in negative fitness effects, especially in nature with its extensive environmental fluctuations. On the other hand, it must be remembered that even significant reduction [50] or extreme increase [26] in the level of crucial bacterial chaperones, such as GroEL/S, leave the cells viable under the laboratory conditions.

In conclusion, both the genetic data and molecular models suggest that chaperone-mediated masking of protein structural instability in bacteria is possible. However, the activity of molecular chaperones appears fine tuned to avoid interference with the protein quality control and multiple other processes. It is not yet possible to estimate how frequently the chaperone-mediated phenotypic masking actually works. However, it appears legitimate to postulate that in bacteria molecular chaperones may occasionally facilitate fixation of a partially deleterious mutation by alleviating its negative phenotypic effects. This may involve transient or sustained increase in the cellular level of chaperones, but the associated costs need not be a long term evolutionary handicap if populations are effectively small, making selection relatively inefficient.

## Molecular chaperones and phenotypic buffering in eukaryotes

The problem of molecular overcrowding is no less acute in eukaryotes. Eukaryotic chaperones are to a large extent homologous to those known in bacteria, although they are more numerous, diverse, and specialized. In particular, they probably constitute two major networks, one active at the time of stress, and the other associated with the production and maturation of new proteins. Accordingly, expression of the former is correlated with reaction to stress and that of the latter with the intensity of translation [57]. This article focuses on the network associated with protein synthesis because these chaperones work throughout most of the lifetime of eukaryotes and therefore are more relevant to phenotypic masking of mutations. As in bacteria, there are cytosolic Hsp70 chaperones that bind elongating polypeptides already during translation. After their synthesis is complete, some proteins are released by the chaperones and ready to function. Others remain bound to the Hsp70 molecules which direct them to more specialized folding machines, e.g. TRiC (a ring-shaped octamer, homologous to bacterial GroEL) or Hsp90. It appears that the folding of a nascent protein is continuously escorted by molecular chaperones, beginning with the moment when its N-terminus leaves the ribosome's tunnel and ending when its final or nearly final three-dimensional structure is formed [18]. Indeed, most destabilized proteins must be sequestered by the molecular chaperones because there are relatively few unfolded or partially unfolded polypeptides in the cytosol [58]. Consistent with this, it has been found that at most a few percent of polypeptides are degraded shortly after synthesis, suggesting that most are protected from proteolytic enzymes [59]. Thus, at least in the eukaryotic cell, the newly synthesized and secondarily destabilized proteins do not form a sizable pool of free molecules that is constantly bound and released by the chaperones. Rather, they are passed along defined pathways of assisted folding [18]. The question is whether the chaperones participating in these pathways work to bring a mutated protein to a structure that is functionally active even if its stability is reduced.

**Figure 2:**
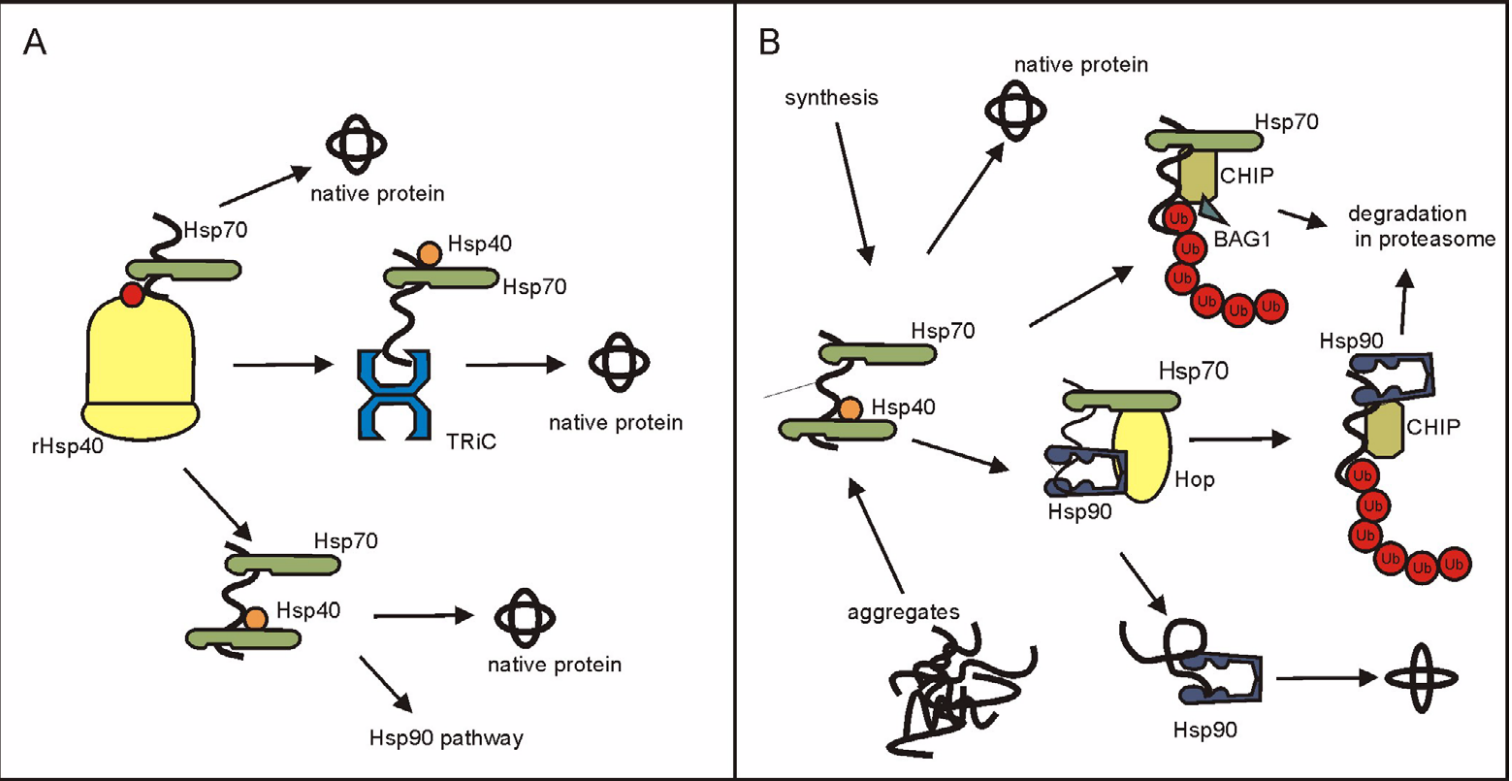
**Chaperone-assisted folding of proteins in eukaryotes [34].(A) **Early phase of folding of newly synthesized proteins. Hsp40 molecules attached to ribosomes (rHsp40) activate cytosolic Hsp70 molecules and direct them to elongating polypeptides. This co-translational chaperoning may be sufficient for the correct folding of many proteins. Folding of some others is assisted by Hsp70 also post-translationally. This can require collaboration of Hsp70 (or prefoldin, not shown) with TRiC. Finally, Hsp70 can direct some proteins to Hsp90, which initiates the late phase of folding. **(B) **Late phase of protein folding and protein quality control in eukaryotes. A protein held by Hsp70 can be released and free from further assistance of the chaperones providing that it manages to adopt a proper conformation. In case of excessively unstable proteins, Hsp70 can recruit the co-chaperones CHIP and BAG1 and this eventually leads to protein degradation in the proteasome. Some special proteins are transferred from Hsp70 to Hsp90 with the help of the co-chaperone HOP. Successfully transferred protein is held by Hsp90 which enables its final modification; alternatively, the protein is directed to proteolysis. The binding by Hsp70 is typical both for newly synthesized and secondarily misfolded polypeptides. If the latter happens, the binding of Hsp70 to polypeptides is governed by Hsp40 chaperones other than those attached to ribosomes. This is one way to differentiate the specificity and intensity of the eukaryotic chaperone activity between normal metabolism (protein synthesis) and stress reaction (protein reactivation).

The folding of proteins in the eukaryotic cell can be divided into an early phase, common to and similar for many proteins, and a late phase, constrained to special types of proteins (Fig. 2A). The early phase starts during translation when the Hsp70 chaperones bind an elongating polypeptide chain. This phase is functionally separated from other chaperone activities because it is initiated by specialized Hsp40 chaperones that are bound to ribosomes and direct Hsp70 molecules to a nascent protein [60]. In yeast, both the co-translationally acting Hsp40 and Hsp70 are distinct from other proteins of these types present in the cell [61,62]. Deletion of genes coding for these proteins does not affect the yeast cell's viability. This raises the question of what is the phenotypic expression of mutations when the co-translational system of chaperones is or is not active. This question is especially important because the chaperones participating in translation are likely to bind many if not all newly synthesized proteins [57]. To answer this, an array of thermally sensitive (protein destabilizing) mutations in different genes was tested. The expectation that the phenotypic effects of protein malfunctioning will be more severe in the chaperone-deficient cells was not confirmed [63]. This result falsifies the hypothesis of chaperone-mediated masking of mutational effects in its strongest form, stating that virtually all chaperones able to bind unwound proteins will in effect support their proper folding [64].

The phenotypic masking of mutations was extensively studied in late-folding chaperones. In eukaryotes, some proteins, including those participating in cellular signaling and regulation, require the assistance of the Hsp90 chaperone in order to finish their folding and to attain a functionally active structure (Fig. 2B) [65]. Hsp90 can be partially inactivated by geldamycin. In fruit flies and plants, such treatment results in phenotypic differentiation of individuals whose parents, although phenotypically alike, are genetically different. The new phenotypic variants can be fixed in selection experiments. Some do not need further pharmacological stimulation to endure [12,28]. In this way, the basic assumptions of evolutionarily relevant phenotypic masking have gained support. First, genetic variation can be covered up under a common phenotype through the activity of the Hsp90 chaperone. Second, the variation can escape the masking effect of the chaperone (in natural environments this may be accomplished by stress) and serve as raw material for heritable adaptation. These findings, comprehensive at the level of genetic analysis, have not been further supported by deciphering the underlying molecular mechanisms [29,37,64,66]. It is also worth noting that some other experiments with fruit flies have shown that the buffering effect of Hsp90 is neither as universal nor as strong as originally believed [67,68]. To further discuss this issue, it is necessary to introduce the concept of protein quality control in eukaryotic cells.

## Chaperones in eukaryotic protein quality control

The native structure of a protein is only one of a great number of conformations that can be adopted by a polypeptide extending over hundreds of amino acids. Non-native spatial structures of non-mutated polypeptides generally occur in two situations, during and shortly after synthesis, and after the destabilization of a mature protein. Protein structural stability may also be altered because of errors arising at transcription or translation. Damaged proteins are marked for disposal by ubiquitylation and then degraded by proteasomes [69]. The accuracy of the cellular systems recognizing incorrect structures and their ability to discriminate between transiently misfolded wild type chains and those permanently changed by mutation are still insufficiently known. Of special interest is the proposition that the molecular chaperones can cooperate actively with the ubiquitin/proteasome system in selecting structurally unstable proteins for degradation [31,32]. If true, this would mean that eukaryotic chaperones do not invariably support the functioning of the defective proteins, including those mutated.

A well studied example of cooperation between molecular chaperones and a protein degradation system is the quality control of proteins in endoplasmatic reticulum (ER). Apart from chaperones specialized in the control of the ER-specific protein modifications, there exists a chaperone system attending the primary control of general stability. In mammals, it is based on BiP, a member of the Hsp70 family. This protein binds nascent and newly synthesized proteins after their co-translational translocation through the ER membrane and assists folding in the lumen. BiP recognizes a variety of proteins and apparently interacts most intensely with slow or atypical folders [70]. Correctly folded proteins proceed to further modifications and export, defective ones are retained in the ER and then are unfolded and ubiquitylated while being translocated through the membrane on their way to cytosolic proteasomes [30,71,72]. It has been shown for a series of thermally sensitive mutants of ER proteins that the intensity of their eradication increases with greater instability of the mutants [73,74]. Thus, these molecular chaperones sense the folding problems, cannot overcome them, and facilitate the degradation of the troubled proteins.

The Hsp70 chaperones serve as detectors of general folding problems also in the eukaryotic cytosol. Normal proteins experience routine binding by the chaperones; in such cases, a likely scenario is that co-chaperones called 'nucleotide exchange factors' will help to release timely the protein from the chaperones [43,75,76]. Proteins that exhibit substantial structural instability are likely passed from the chaperones to proteases. For example, a mutated form of CFTR (cystic fibrosis transmembrane conductance regulator) shows prolonged interaction with the cytosolic Hsp70 system which probably constitutes a signal for its ubiquitylation and degradation [77,78]. Covalent attachment of ubiquitins is performed by ubiquitin-conjugating enzymes, E2, and ubiquitin ligases, E3 [69]. E3 ligases are especially important for protein quality control because they are responsible for selection of polypeptides destined for ubiquitylation. It has been shown that one of such ligases, CHIP, is effectively a co-chaperone of Hsp70. It docks to the Hsp70 chaperone that holds a misfolded protein, recruits an E2 enzyme and triggers the ubiquitylation of the protein. Two further steps are performed by a single protein, BAG-1, another co-chaperone of Hsp70. It links the chaperone to a proteasome and releases the ubiqitylated polypeptide so it can be transferred from the former to the latter [31,32]. The whole model is impressively complete in its functional and structural aspects. Understandably, it serves as the chief example of cooperation, instead of competition, between cytosolic chaperones and proteases.

CHIP-mediated protein quality control and degradation has been demonstrated for several different proteins [32]. Still, it is not clear whether most destabilized proteins are detected and disposed in this way. Notably, there is no known homologue of CHIP in the budding yeast which therefore must apply a different interface between the chaperones and the proteases. Recently, a new example of a cytosolic chaperone-mediated system of decision making concerning folding or degradation has been described. The folding of a newly synthesized mammalian cytosolic protein, VHL tumor suppressor, requires that Hsp70 cooperates with TRiC. When the folding is defective, Hsp70 transfers the protein to a chaperone complex that includes Hsp90 and this eventually leads to degradation of VHL. The two pathways are distinct: TRiC is required for folding but dispensable for degradation, the reverse is true for Hsp90. Hsp70 is at the crossroads, its activity is necessary for both functions [79]. This system, and that dependent on CHIP, have much in common. Both assume that a troubled protein is first recognized and bound by the Hsp70 chaperone and that the outcome of this event depends on subsequent interactions between the protein, Hsp70, and other chaperones and co-chaperones.

**Figure 3:**
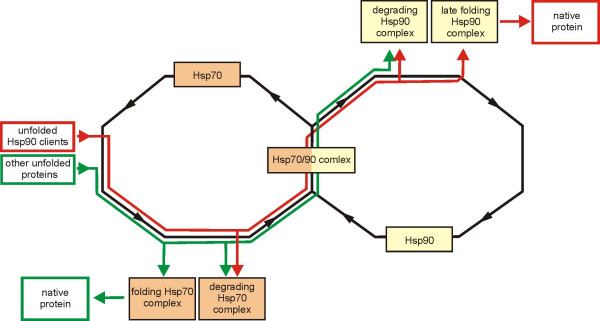
**Substrate selection and processing in the eukaryotic Hsp70/Hsp90 chaperone system.** Newly synthesized or secondarily unfolded proteins are recognized and bound by Hsp70. These can be either proteins that require assistance of Hsp90 at later steps of their folding or any other polypeptides. The latter (green line) can attain their native configuration with the assistance of the folding Hsp70 complex [18]. or be disposed with the help of the degrading Hsp70 complex if they are excessively unstable [34]. It is also possible that proteins that normally do not need to interact with Hsp90 in the process of their folding are nevertheless passed to the degrading Hsp90 complex when they have to be disposed [79]. Proteins that do require Hsp90 for the late steps of their folding (red line) can be directed to degradation before contacting this chaperone if they are excessively unstable [31]. Otherwise, they are passed from Hsp70 to Hsp90 through an intermediate complex containing both these chaperones. Maturation of these proteins can be finished in the late folding Hsp90 complex [82] or aborted in the degrading Hsp90 complex if they do not follow expected scenario of folding [84]. Molecules which need to be assisted by Hsp90 but are dropped before their fold is completed have to re-enter their path of chaperone-assisted maturation primarily, or perhaps exclusively through binding to the Hsp70 molecules [83]. This renders them to another round of quality control.

It is likely that other chaperone systems also participate in the broadly defined quality control of proteins. It has already been mentioned that an experimental attempt to demonstrate that the yeast co-translational Hsp40/70 system can support the functioning of mutationally destabilized proteins was unsuccessful [63]. This result becomes comprehensible when protein quality control is taken into account. The Hsp70 chaperones are present on the pathways leading either to protein folding or protein disposal. Apparently, proteins that contain mutations causing defects large enough to produce thermo-sensitive phenotypes are directed mostly to the second path (Fig. 3). Indeed, ongoing experiments show that the in vivo activity of mutated proteins is actually less efficient when the Hsp40/70 system is active. This suggests that the chaperones participate in sorting out the defective molecules (Tomala and Korona, unpublished). It has been postulated that the general purpose of co-translational chaperones is to enhance the efficiency of protein folding [18]. However, it is also plausible that not low folding efficiency but the danger of formation of toxic misfolded and/or aggregated species is the ultimate reason why the newly synthesized proteins are chaperoned [57,80]. In both cases, but especially in the latter, the co-translational chaperones would rather expose than conceal genetic defects because their function would be to scrutinize the folding of gene products which would result in preferential elimination of mutants.

The quality of proteins maturing in the ER is strictly controlled both in terms of general stability and correctness of specific modifications [72,81]. The hypothesis of phenotypic buffering of mutational damage by the molecular chaperones has never been explicitly applied to these proteins. Therefore ER proteins, comprising a large share of all proteins present in the eukaryotic cell, will not be further taken into account as potential subjects of the mutation-masking activity of chaperones.

## Direct and indirect effects of Hsp90 activity

Hsp90 is a chaperone engaged in late phases of protein folding. Substrates of Hsp90 are already partly folded; many of which are proteins awaiting final conformational changes making them active as signal transducers [65]. The standard role of Hsp90 is to bind and maintain these proteins in a conformation that enables the required modification to be carried out [82]. At the time when the concept of Hsp90-mediated masking was born, it could be assumed that Hsp90 performs its function by direct and recurrent binding and release of the assisted protein and that such activity would be most desirable if the protein was mutated [12]. The current understanding of substrate acquisition by Hsp90 is markedly different. A typical protein requiring the assistance of Hsp90 is first bound by Hsp70 and only then transferred from Hsp70 to a Hsp90-centered complex [82,83]. There is no indication that this pattern does not apply to proteins allegedly engaged in phenotypic masking. Thus, Hsp90 client proteins would not be exempt from the quality control typical for the substrates of Hsp70 (Fig. 3). Furthermore, it is difficult to imagine that a protein affected by mutation is chaperoned in a special way in the Hsp90 complex, if it gets into it, to increase the chance for required modification [35,83]. It was mentioned above that atypical substrates of Hsp90 may be instead directed towards degradation [79].

A revealing example of how Hsp90 actually aids protein quality control has been reported recently. Hsp90 is needed at a certain stage of maturation of CFTR. The folding of a mutant CFTR is often terminated by the quality control process described above, mediated by Hsp70 and CHIP. If the defective CFTR is nonetheless faced with Hsp90, it is not yet safe. Hsp90 has a sequentially changing complement of co-chaperones. They determine the scenario of folding and the structure/behavior of a protein must conform to the expected pattern to allow efficient co-operation. Some mutant CFTR fall outside the range of tolerable incongruity and therefore the composition of co-chaperones of Hsp90 shifts towards factors promoting ubiquitylation and degradation [84]. It is possible that a similar evaluation is carried out for different mutations in different proteins and that at least some are subject to chaperone-assisted degradation. This was demonstrated for mutations with relatively strong destabilizing effects, but there is no indication that less affected proteins are not disposed in this way, although probably at a lower rate. In sum, the general idea that Hsp90 ameliorates phenotypic expression of mutations by directly assisting the folding of destabilized proteins becomes increasingly incompatible with current molecular models (Fig. 3).

This conclusion is not contradicted by the experimental findings that phenotypic differentiation of genetically variable organisms increases when the activity of Hsp90 decreases. Hsp90 can have a few hundred client proteins [65]. The hypothesis of phenotypic masking was originally focused on mutations residing in these proteins. However, the direct clients of Hsp90 are critical for functioning of many pathways in which an even larger number of proteins are engaged. Malfunctioning of Hsp90 will result in malfunctioning of its client proteins even if the latter are not mutated. This, in turn, will jeopardize functioning of multiple pathways, especially those containing mutated and therefore less efficient proteins [64]. In this way, the proper functioning of Hsp90, and other chaperones, may buffer phenotypic expression of mutations even if the chaperones do not physically interact with the mutated proteins. "Mutated" need not mean destroyed. Heritable variants of proteins whose functioning depends directly or indirectly on Hsp90 can give different phenotypic traits whenever the cellular level of this chaperone falls below some critical threshold. In this way, activity of Hsp90 buffers phenotypic expression of genetic variation [29,66]. It has been claimed that the dependence of phenotypic variation on the activity of Hsp90 makes this chaperone a very special capacitor of evolution [28,37,66]. However, "release" of phenotypic variation in response to malfunctioning of a gene is ubiquitous and not restricted to Hsp90. For example, malfunctioning of transcriptional factors also 'uncovers' genetic variation because such defects are likely to lead to extensive and genotype-dependent changes in expression of genes. Furthermore, altered functioning of chromatin remodeling enzymes may uncover not only genetic but also epigenetic variation [85]. More generally, malfunctioning of any gene interacting with many other genes is likely to increase phenotypic variation [86].

Molecular chaperones are included among the mechanisms promoting stabilization because they support multiple links among proteins and in this way add to the overall robustness of genetic networks [87]. However, it is purely speculative to propose that an important factor governing the evolution of molecular chaperones is the need to increase the capability of organisms for the rapid remodeling of phenotypes at a time when evolutionary novelties are needed. According to this view, Hsp90 could be considered a regulator of evolvability because it can modulate the expression of genetic variation and in this way hide or expose mutations to natural selection [66,88]. It has been recognized that one evolutionary cost of this mechanism is the potential accumulation and occasional exposure of a number of mutations which would very often be harmful rather than advantageous [e.g. [37,66,88]]. However, any compromise in the accuracy of protein quality control would bring another effect, that is, impaired elimination of all damaged proteins, including those produced by non-mutated genes. Overcrowding and translational errors can-not be avoided. This cost would not be stochastic and would not increase variation. Instead, it would lower the fitness of all individuals in a population and would not promote the origin of hopeful variants. This is another reason for caution when suggesting that molecular chaperones may serve as modulators of evolvability. The sensitivity of organisms to impairment of protein quality control, especially long-lived ones, is increasingly well recognized in biomedicine.

## Molecular chaperones and disease

The search for links between phenotypes and molecular mechanisms is especially difficult if complex traits, such as fitness or health, are considered. For example, it was found that the overexpression of Hsp70 in fruit flies suppresses neurodegeneration caused either by proteins with expanded polyglutamine tracts or mutated α-synuclein [89,90]. In both cases the beneficial role of the Hsp70 chaperone probably does not result from the restoration of the functional structures of the proteins but from a reduction of cytotoxicity caused by protein aggregation. On the one hand, this is an example of a phenotypic cover-up mediated by molecular chaperones. On the other, these phenotypically masked mutations probably have little bearing on future adaptation. Other examples come from studies on the role of Hsp90 in cancer development. The pharmacological inhibition of this chaperone may lead to successful treatment [91]. Interpretation of this finding is not straightforward. It has been hypothesized that cancer cells harbor a large amount of mutated proteins and therefore their functioning is dependent on the ability of Hsp90 to buffer multiple genetic damage [92]. However, the repression of Hsp90 means that not only mutated oncogenic proteins but also a number of non-mutated signal proteins are directed towards degradation instead of maturation [93]. A further complication is that the transformed cells are faced with a stressful tumor microenvironment owing to hypoxia, nutrient deprivation, and acidosis [94]. The most important effect of the repression of Hsp90, whether it is the unmasking of mutations, disturbance of stress response, or general deregulation of cellular signaling, is too complex to pinpoint at present [95]. Again, the potential adaptive value of mutations associated with cancer and possibly masked by Hsp90 is at best doubtful. We suggest that the analysis of the role of chaperones at the level of whole cells or organisms is hardly possible considering the present state of theory and technology. Careful examination of direct interactions between molecular chaperones and their 'clients' is probably a more promising way to understand how molecular chaperoning shapes the evolution of proteins.

## Chaperones and selection against mutations

Only a small fraction of single substitutions change the final structure of a protein so extensively that it becomes non-functional. Many more lead to protein variants whose final structure is little changed but stability is violated [96-99]. The severity of observable phenotypic effects can vary extensively. A mutation has to cause a relatively strong, thermally dependent destabilization to result in a good conditional (ts, thermally sensitive) phenotype of practical laboratory value [100]. The remaining mutations may have smaller phenotypic effects but are nevertheless susceptible to the purifying action of natural selection. The strength of natural selection is of course limited, i.e. the complete eradication of continuously arising spontaneous mutations is not expected [101]. By magnifying the decrease in the cellular level of mutationally destabilized proteins, the chaperones would make such selection more effective. This would lead to a reduction of genetic polymorphism in populations. Insufficient genetic variation can limit the rate of adaptation at the time of environmental change [102]. Referring to the rate of molecular evolution, chaperones, at least in some cases, may slow it down by augmenting the phenotypic effects of amino acid substitutions and thus making more likely that the mutations would rather be purged by selection than fixed by drift. This perspective is clearly different from that postulating that chaperones are capacitors of evolution because they typically mask phenotypic expression of mutations and thus maintain a reservoir of variation needed for the creation of novelties [12,28,37,66,88]. At present, experimental evidence for the decrease of the deleterious effects of mutations as a direct result of chaperone activity is restricted to bacteria under specific laboratory arrangements [49,50]. Evidence for augmentation of mutational harm by the direct activity of chaperones is also restricted to a few experiments, although it is found both in prokaryotes and eukaryotes. Any claims about the domination of the former or the latter outcome would be premature at present.

## Conclusion

Current understanding of protein folding and quality control is admittedly unsatisfactory and based on a limited number of examples. However, the perspective adopted here is supported not only by the discussed cases, but reflects a major conceptual shift in research on the cellular role of molecular chaperones. It was first established that the heat shock proteins are also active under normal metabolism and that different classes of the hsps have generally similar activity at the molecular level [103]. This leads to a model in which a nascent or destabilized protein benefits from assistance of different chaperones, each contributing more or less individually and additively to the process of folding [104,105]. In this scenario, the chaperones are mostly helpers. It was then gradually recognized that the chaperones function often, if not mostly, in assemblies which have their own structure and dynamics [18,30,32,57]. The assistance provided by these ordered consortia of chaperones appears conditional, because incompatibility with the prescribed folding programs can trigger redirection to degradation [72,84]. In this way, the quality control of proteins, especially large and complex proteins, may be extended over several steps of folding and maturation. This means an additional and possibly important role of the chaperones as examiners. The number of chaperone-assisted folding pathways is probably limited, with many proteins coming through each of them. Participation in the same pathway means sharing the same folding environment. Thus, broad classes of proteins meet similar requirements and this can shape their evolution. A possible future direction in research is to identify particular constraints or relaxations that are created by different groups of chaperones. This would be a truly comprehensive approach to the study of the evolutionary role of molecular chaperones.

## Reviewers' comments

### Reviewer's report 1

A. S. Kondrashov, University of Michigan

This is a comprehensive review of a very interesting subject. How chaperones affect evolution in general and selection against deleterious mutations in particular? The answer depends on what chaperones are doing molecularly. Do they mostly help proteins to fold correctly, or do they mostly cause degradation of proteins that have some structural defects? The data are controversial and, as far as I can judge, the review comprehensively covers this field. Because the paper mostly deals with molecular mechanisms, and I am an evolutionary biologist, I really have only two comments.

(1) Within the traditional paradigm of chaperones as "helpers", there still is a radical difference between chaperones ameliorating impacts of deleterious mutations and chaperones as capacitors of further evolution. Natural selection can easily explain the first situation, but not the second one. Indeed, an allele that increases fitness of individuals that carry a particular set of deleterious mutations, because the affected proteins fold better, will be selected for. In contrast, it is not clear how selection can favor an allele that increases the potential of the population for evolution in the future. Apparently, the only way this can happen is through group selection, which is unlikely to play any major role in nature.

Of course, one can argue that ameliorating impacts of deleterious mutations will inevitably lead to increase of the standing genetic variance and to more opportunities for evolution on the future. The first of these statements is certainly true, but the second may be wrong. The key issue is: how often a mutation that is currently deleterious may become advantageous in not-too-distant future? In other words, are deleterious mutations deleterious unconditionally or many of them are conditionally beneficial? Conventional wisdom of genetics seems to favor the first possibility, although there may be exceptions. However, this issue, which is central for our understanding of evolution, is still far from being settled.

(2) If, in contrast, chaperones mostly work as "protein quality control", the reason for their evolution must be removal of accidentally misfolded proteins and, perhaps, proteins misfolded due to somatic mutations. In both cases, the action of chaperones must increase fitness of a particular genotype. Indeed, it is hard to imagine how selection can favor an allele that make selection against germline mutations more stringet, by reducing fitnesses of a mutation-carrying genotypes.

### Reviewer's report 2

J. Höehfeld, University of Bonn, Germany (nominated by A. Eyre-Walker, University of Sussex, Brighton, UK)

In their manuscript Tomala and Korona propose a significant extension of previous concepts regarding the role of molecular chaperones in evolution. Chaperones are currently widely viewed as capacitors of protein evolution because their intrinsic activity as facilitators of protein folding seems to enable them to maintain the function of mutationally destabilized proteins and, therefore, provides a means to mask or buffer the phenotypic expression of mutations. Tomala and Korona now argue for a re-evaluation of these functional concepts in particular in the light of recent findings that point to an active involvement of molecular chaperones in protein degradation. The findings suggest that chaperoning of a mutationally destabilized protein may not necessarily lead to the adoption of a functional protein structure but may result in the removal of the damaged protein by degradation as part of the cellular protein quality control. Tomala and Korona summarize in a comprehensive manner recent biochemical and cell biological findings regarding the cooperation of chaperones with protein degradation systems and then discuss the relevance of these findings with regard to the proposed role of chaperones in evolution. As such the manuscript represents a very interesting and stimulating contribution to this area of research and should be appealing to a broad range of researchers.

However, some aspects need further clarification. The authors very strongly argue against previous functional concepts not only on the basis of the recent findings regarding chaperone/proteasome cooperation, but also often point to mechanisms that would not involve direct interactions between chaperones and mutationally destabilized proteins. The authors mention, for example, that a multitude of signaling pathways is dependent on the activity of the chaperone Hsp90 and accordingly pharmacological inhibition of Hsp90 may result in a pleiotropic disturbance of cell functions, resulting in the phenotypic unmasking of mutations without involving a direct interaction with the mutated proteins. Importantly, this would argue similarly against a role of the chaperones as folding factors, rescuing the mutated proteins, and against a role as facilitators of protein degradation, removing the protein garbage, as the molecular basis for phenotypic buffering. Therefore, it seems that the manuscript should be restructured to separate these two aspects more clearly (direct interactions involving either folding or degradation versus indirect effects).

If the authors consider chaperone-assisted degradation as an important aspect also chaperone-assisted folding and rescuing should be of relevance, because we know very little about the mechanisms that determine the eventual outcome of the chaperone/substrate interaction – being it degradation or folding. In this regard the assessment of previous work pointing to the role of chaperones as capacitors of protein evolution seems to be overly negative. What is required is probably not a complete reconsideration of underlying molecular mechanisms but rather an extension of previous concepts, which incorporates chaperone-assisted degradation. Chaperone-assisted degradation of a mutationally destabilized protein could apparently prevent phenotypic expression because loss of protein function often occurs without consequences for viability. Mutations could accumulate on the level of the DNA and could be unmasked when chaperone activity declines for example under conditions of environmental stress. This would further emphasize the role of chaperones as capacitors of protein evolution – not invoking solely a function as folding helpers – but considering a broader role as protein examiners.

#### Minor issues

Paragraph 'Phenotypic masking revisited': The sentence 'the yeast co-translational Hsp40/70 system does not support functioning of mutationally destabilized proteins' seems to be rather bold. Do the available experimental data really justify this conclusion?

Regarding the increased sensitivity of cancer cells against Hsp90 inhibitors the authors state that it is not clear, why the scarcity of Hsp90 is more damaging to cancer cells that to their non-transformed counterparts. It has been observed, however, that Hsp90 in cancer cells displays a higher affinity for inhibitory drugs, resulting in the described differences in sensitivity

##### Authors' response

*In line with the reviewer's suggestions we now separate more clearly the direct effects of chaperone activity (stability of proteins) from indirect ones (stability of genetic networks). This distinction is mentioned in the abstract and then discussed in a special chapter on direct and indirect effects of Hsp90 activity. Indeed, the malfunctioning of multiple metabolic pathways whose members depend on Hsp90 is likely to be detrimental for many cellular processes and protein degradation is not an exception. This is what we describe it as an extensive and largely deleterious pleiotropy. We propose, however, that a spontaneous change or experimental adjustment of chaperone activity aimed at saving rare mutants would likely result in decreased quality control of non-mutated proteins and the ensuing danger of protein misfolding and aggregation. The costs can be potentially high, especially for long-lived organisms. This is why we hesitate to accept the concept of Hsp90 as a capacitor of evolution*.

*While writing about 'reconsideration' we meant broadening, not abandonment of previous concepts. This should be even more clear now after introducing a new example of chaperone-mediated alleviation of deleterious mutational effect (ts mutation of a phage coat protein – suggested by Dr Drummond). We now explicitly write that a few (one or two) but genuine examples of alleviation of mutational damage through intensified interaction with chaperones can be found. Admittedly, examples of chaperone-mediated disposal of mutated proteins are also scarce. Therefore in both the former and the present manuscript we do not try to answer the question whether chaperones participate mostly in rescue or in destruction of mutated proteins. Our intention was not to "strongly argue against previous functional concepts". We did concentrate mostly on the involvement of chaperones in protein quality control but this was because this topic was insufficiently covered in the past. We do not mean the specialist literature on chaperones and proteolysis but journals on evolutionary biology*.

*The suggestions expressed in 'minor issues' have been accepted*.

### Reviewer's report 3

D. A. Drummond, FAS Center for Systems Biology, Harvard University (nominated by C. Adami, California Institute o Technology, Pasadena)

The central thesis of this work is that phenotypic masking of mutations by chaperones has no direct empirical support when the evidence is carefully reviewed. Results from the Teschke lab directly contradict this position as described below. Taking the abstract at face value, the authors' program comes across as largely an attempt to discredit phenotypic buffering, which is unfortunate as they provide a valuable review of chaperone systems in the course of the paper. The work is elegantly written and polished, and should simply be refocused as a review which places phenotypic buffering in perspective rather than attempting to dismiss it.

In my reading of the literature, both supporters and detractors of the phenotypic buffering hypothesis accept that chaperones have a wide variety of roles, and supporters do not claim that phenotypic buffering is the primary activity of any chaperone. The strong argument the present authors seem to dislike is the claim that all chaperones can rescue mutation-destabilized proteins to some degree. They make an excellent case for many distinguishable roles for chaperones which cannot be reduced to such a single common denominator, and as an answer to this "strong buffering principle" the present work is welcome. Nevertheless, I do not think the strong principle is widely credited.

The weak version holds that chaperones sometimes act to rescue mutated proteins. Against this weaker argument the authors's case is not particularly compelling. Consider the circumstantial evidence. The authors admit throughout the manuscript how plausible it would be for chaperones to rescue mutated proteins; it is difficult to disagree. Nonetheless, faced with the finding that mutated E. coli lines grow better when chaperones are abundant, and that other mutated lines have concomitantly increased chaperone levels, the authors argue ("Molecular chaperones and phenotypic buffering in bacteria" section, paragraph 2) that some mutations must have lowered the expression levels of proteins resulting in rate-limiting metabolic throttles, and some of these proteins must require chaperones for maturation or remodeling. While this scenario is possible, I do not find it convincing at all. "Expression-decreasing mutations yielding rate-limiting abundance changes in proteins that require chaperoning for maturation or remodeling" seems a much more restrictive (read: improbable) set than "destabilizing mutations in coding sequences."

More convincingly to the authors, Van Dyk et al. Nature (1989) reports that many ts alleles in S. typhimurium are suppressed by overexpression of GroEL/S (not GroEL alone as Tomala & Korona report). The present manuscript admits, "Because heat sensitivity typically accompanies protein instability, this result is probably the closest to demonstrating that chaperones can restore the functionality of mutationally destabilized proteins."

A better example comes from Nakonechny and Teschke (JBC 1998) who show that, in vivo, 1) ts mutants of phage coat protein are rescued by GroEL/S overexpression; 2) wild-type GroEL/S supports plating efficiency (phage titer, a measure of phage fitness) substantially better than mutated versions of GroEL/S even at permissive temperatures; and 3) GroEL binds directly to the destabilized coat proteins more than wild-type coat proteins as assessed by co-IP, increasingly so as a function of temperature. Teschke's group has also carried out elaborate experiments which establish that these ts mutants are indeed destabilized (S.M. Doyle et al. JBC 2004).

The Teschke-group results represent a clear, complete story of mutant instability, chaperone rescue by direct interaction, and phenotypic buffering that has a direct impact on fitness (in this case, of the phage). This story directly contradicts the thesis of the present review. As a case in point, these results answer the concerns used to close the relevant paragraph: "Either phenotypic observations are convincing but molecular interpretation unsure, or molecular mechanisms are nearly pinpointed but their fitness effects unknown." I recommend that sentence be removed and the Teschke results reported; as stated above, the central thesis of the paper should be modified.

The related statement that chaperone overexpression may impose fitness costs ("this study did not test for possible negative fitness effects associated with the upregulated expression of chaperones") comes across as sleight-of-hand – the question is first whether chaperones can buffer mutations, and only then if they can do so without harming the organism in other ways. The phenotypic buffering hypothesis covers cases in which chaperones buffer a mutation in an essential gene without being upregulated. Perhaps the authors would argue that this is impossible, but it is not obviously so (consider a mutation in an essential, low-expression protein which, in the absence of chaperones, would lead to kinetic trapping during folding, but which is relieved in the presence of wild-type levels of chaperones). As long as chaperones can render neutral such mutations which are otherwise deleterious, those mutations will go to fixation with a probability determined by population size instead of by their deleterious effect, and the chaperone will have effectively buffered the mutation in a way that contributes to evolutionary variation.

The authors' point that phenotypic buffering by Hsp90 has yet to be directly established is well-taken. Fixation on Hsp90 proceeds from the original Rutherford and Lindquist paper, but it must be emphasized that the importance of that paper derived largely from the observation of morphological variants in the absence of chaperone – contrast this with loss of function in the Teschke coat-protein mutants. (One may rightly ask whether the morphological variants found have any evolutionary significance, or are just the organismal equivalent of misfolding.) If the authors wish to focus on Hsp90 studies, they have a case; still, the possibility of phenotypic buffering by any chaperone seems to be the broader and more important question.

The authors improperly overlook the role of translation errors as by far the dominant source of mutated, destabilized proteins in the cell and therefore a major relevant substrate of chaperones. Translational missense errors occur at a rate between 1e-3 and 1e-4 per codon (Ogle and Ramakrishnan Ann Rev Biochem 2005), so that roughly 20% of molecules of an average-length (~400-residue) protein are expected to contain at least one missense error. For long proteins such as dynein or titin, it is likely that most protein molecules contain at least one error. Mutated proteins in the cell must therefore expected to be common under normal physiological conditions. It seems plausible that many chaperones exist, in part, to rescue these non-heritable mutants from the premature aggregation and kinetic traps to which they are prone, so that they may go on to be productive members of cellular society. In this view, phenotypic buffering is simply an inevitable side-effect – chaperones, after all, cannot tell which mutant proteins arose because of a DNA mutation or a translation error. An argument that chaperones exist *in order to* buffer genetic variation cannot be supported by this model, but I am unaware of such claims.

At any rate, I suggest that the present authors devote more time to discussing translation errors, perhaps using C.M. Grant et al. Mol Micro 1989 as jumping-off point, where it is shown that elevated mistranslation induced by paromomycin induces the heat-shock response in yeast.

As suggested above, the authors may wish to consider refocusing their review. My reading of the authors' evidence and the literature yields a rather more accommodating view than the present manuscript espouses. In short, the optimal strategy for the cell is to save (buffer) proteins which can be saved, and degrade those which cannot. Consider only thermodynamic stability as a narrow example. No amount of chaperoning can rescue a thermodynamically unstable protein. But because mutations induce a wide range of free energy changes, some mutations will terminally destabilize a protein (so that it must be disposed of), some will marginally destabilize it (so that it needs chaperone help to avoid aggregation or degradation during folding, but no more), and some will leave its stability unchanged or even stabilize it (no worries!). An elaborate, connected system of chaperones must triage these patients, with the outcome optimally depending on the severity of the wound. Different chaperones may play different roles, but it must be beneficial to save some patients. (The authors emphasize that the reason to dispose of a protein may be to prevent formation of a toxic misfolded species rather than preserve a functioning protein, an argument I find very convincing and one that equally supports assisting the folding of some otherwise wobbly molecules.) The major questions concern how, and how well, this blind, stochastic triage system categorizes its substrates, which chaperones play which (presumably overlapping) roles, and how the system can be recoverably or irretrievably perturbed.

#### Authors' response

*We never claimed that genuine buffering through direct contact between mutated proteins and chaperones is impossible. Since the reviewer cites several of our sentences, we would also like to cite one: "In conclusion, both the genetic data and molecular models suggest that chaperone-mediated masking of protein structural instability in bacteria is possible." The reviewer concentrates on our evaluation of experiments with bacteria. First, we uphold our opinion that experiments involving a large number of unknown mutations are difficult to interpret. In such situations, low levels of chaperones may mean insufficient chaperoning of many unstable proteins. Alternatively, a synthetically harmful effect may be produced through genetic interaction between low level of chaperones and some null mutation(s)*.

*On the other hand, we agree that the case of a thermosensitive mutation in a phage coat protein provides the currently best example of chaperone-mediated buffering of mutational damage. We were not familiar with this example because, as far as we know, it was not mentioned in the literature on phenotypic masking, genetic buffering, evolutionary capacitance, etc. We now cite this work. However, we find it difficult to acknowledge that the question of costs impaired by overexpression of chaperones is less important than the fact that overexpression may bring about phenotypic buffering (under laboratory conditions). The balance between benefits and costs is the critical factor in evolution, especially in large natural populations*.

*We are not aware of previous articles that have called for reconsideration of the evolutionary role of molecular chaperones due to their participation in protein quality control. This function is by far less known than the role of chaperones in stabilization of proteins and genetic networks, at least among evolutionary biologists and geneticists. Our message is not, or not only, that the buffering effect of chaperone activity is rarer than believed. We suggest that the scarcity of such evidence may have a deep functional basis, that is, the dual role of chaperones as both helpers and examiners of proteins*.

*We did mention that translational errors could be a source of misfolded proteins, although very briefly. We now introduce the requested citations and quantitative estimations of the rate at which errors occur. Generally, we make clear that translational errors together with macromolecular overcrowding are likely to be a major source of misfolding of proteins coded by non-mutated genes*.

*We did not suggest that anybody claimed that chaperones exist in order to buffer genetic variation. It is generally agreed that this can only be a secondary function. The question is whether this is only a side effect or a factor in their evolution. The latter would mean that the activity of chaperones is tuned in a way that secures not only their standard functions but also allows for accumulation of genetic variation. We now expand more on this hypothesis by referring to the concept of Hsp90 as a 'general regulator of evolvability'. This argument states that even if the populations (lineages) with increased accumulation of buffered mutations would be less fit over short time scales, they would eventually win because of their supremacy during periods of rapid adaptation. This idea is considered by many as controversial and we add one more argument against it. The danger of misfolding and aggregation of proteins coded by non-mutated genes is probably high enough that lowering the stringency of protein quality control in order to save rare mutants is unlikely. These evolutionary questions, and not a critique of previous experimental work, are the focus of our article. To make it obvious we rewrote the abstract and introduced several other clarifications*.

## Competing interests

The author(s) declare that they have no competing interests.
